# Work Stressors and Occupational Health of Young Employees: The Moderating Role of Work Adaptability

**DOI:** 10.3389/fpsyg.2022.796710

**Published:** 2022-04-26

**Authors:** Houyu Zhou, Quangquang Zheng

**Affiliations:** ^1^Department of Psychology, Jing Hengyi School of Education, Hangzhou Normal University, Hangzhou, China; ^2^Department of Psychology and Behavioral Science, Zhejiang University, Hangzhou, China

**Keywords:** occupation health, work adaptability, young employee, work stressor, moderating effects, China

## Abstract

Work adaptability refers to the work experience, habits, and skills that enable an individual to adapt to current or changing work tasks and situations. It is a coping resource that individuals use to mitigate various types of stress. Adopting the interaction model of work stress, this study investigated 168 young employees in 20 organizations in Zhejiang Province through interview research and a questionnaire survey. The results show that work adaptability has a significant main effect on occupational health. The work adaptability of employees plays a moderating role in the relationship between occupational health and lack of work meaning stress, role conflict stress, interpersonal relationship stress, negative organizational atmosphere stress, and total score of work stressors. Young employees with high work adaptability have worse occupational health under high-level stress situations due to a lack of work meaning. For promoting occupational health in young employees, organizations should have this group of workers complete meaningful jobs or inform them of the importance of their jobs, reduce role conflict, and create a supportive organizational atmosphere. For management, it is imperative to eliminate high-level stress that stems from a lack of work meaning in order to retain young employees with high work adaptability. These findings shed light on how work adaptability helps young employees deal with stress and improve their occupational health. In organizational and self-stress management, it is beneficial to improve employees’ work adaptability continuously as a means of effectively resisting stress and maintaining occupational health.

## Introduction

According to career development theory (Hall, 1980, 1987), young people who have just started their first job are at the career exploration stage. They start developing their own professional outlook and personality, constantly examine themselves, and test various professional roles. At this stage, employees possess an unstable professional personality, and they are not very productive (Lu, 1998; Broverman and Lazarus, 2006). Some researchers believe that people aged 20–30 are at the early career stage, which is a period when employees join an organization and are gradually “organized” and accepted into the workplace (Zhang, 2002). To achieve these goals, young employees need to overcome various pressures and difficulties that stem from their work (Baker and Karasek, 2000; Nejad et al., 2021). Examples of these types of challenges include completing new job tasks, building interpersonal relationships in the workplace, and accumulating work experience (Lazarus and Launier, 1978). Do these pressures and difficulties lead to persistent physical and mental stress among young employees and affect their health? What strategies can young employees who are in the career exploration period use to adjust and cope with their initial work stress and maintain occupational health? Answering these questions is of great significance for organizations that want to manage young employees’ work stress. Additionally, it is of great significance and value to effectively carry out self-stress management for young employees and optimize their career development paths.

In the present study, we focus on the positive role that adaptive resources play in the lives of young employees during the career exploration stage. According to Erickson (2021), individuals are active explorers who can adapt to a different environment and want to control it. Only by knowing the real world can they successfully adapt. Individual development is the result of the interaction between internal instincts and external cultural and social requirements (Ross, 2014). The process of development involves constant adaptation (Nie, 2005). Young employees are typically in the critical development period of fully integrating into a social environment, especially in the context of China’s social environment, which is dominated by collectivism. They can adapt to the environment by participating in various challenging pressure tasks, such as technological innovation, intensified competition, product upgrading, and diverse customer demands (Chen, 2020) and obtaining healthy occupational development. Chinese youths thus make up one of the representative groups suitable for observing the role of adaptive resources.

Among the various factors related to the utilization of adaptive resources, the current research considers the correlation effect between young employees’ work stress events and adaptive resources. According to the transaction theory of work stress, adaptive resources are important tools that come into play when a person is under pressure (Folkman et al., 1987; Lazarus and Folkman, 1987; Avey et al., 2010). Because every individual has different adaptive resources, adaptive responses to work can differ depending on a given situation. For instance, a reaction may be favorable, insufficient, excessive, or inappropriate (Ross, 2014; Rigó et al., 2021). The current research examines the effect of complex interactions between adaptive resources and work stress because doing so can help young employees refer to the experiences of their predecessors, manage their own work pressures and adaptive resources, and achieve a favorable adaptive response state.

Among the individual factors that integrate adaptive resources to effectively cope with work pressure and produce an adaptive response, the current research selected occupational health as the criterion variable. Researchers and practitioners generally believe that occupation is still an important health determinant (Soljak, 2021). Occupational stress has a significant impact on the occupational health of employees (Quick and Henderson, 2016; Wang and Li, 2016), and how well an organization manages the occupational health and work stress of their young employees determines whether their workers are willing to stay (Xiong, 2019; Bai, 2020). Utilizing, integrating, and developing adaptive resources among employees in order to help them manage stress and achieve sustainable, healthy development has thus become an important goal for many organizations (Quick and Henderson, 2016; United Nations, 2018).

A limited number of studies have explored the association between work stress and occupational health in young employees in China (Xiong, 2019; Bai, 2020). This study provides a better understanding of the link between work adaptability and occupational health by examining the relationship between work stress, occupational health, and work adaptability in young employees at the career exploration stage, this study provides a better understanding of the links that exist between work adaptability and occupational health. Moreover, in shedding light on the moderating effect that work adaptability has in the relationship between work stress and occupational health, this study offers strategies that organizations can apply to tackle the complex issue of work stress, improve their young employee’s work adaptability, and promote occupational health. In summary, the aim of this research is to assess whether work adaptability enhances occupational health and examine the moderating effect of work adaptability on the relationship between work stress and occupational health in young employees at the career exploration stage. The outcomes of this study offer both theoretical and practical implications and contribute to the implementation of effective stress management and adaptive resource utilization strategies for organizations and groups of young employees. Theoretical underpinnings and hypothesis development, methods adopted in this research, results and hypothesis testing, discussion, conclusion, limitation of this research, and future direction of this research are presented in the following sections.

## Hypothesis Development

### Work Stress

Work stress, also known as occupational stress, refers to pressure that people experience from professional tasks or roles (Lazarus and Folkman, 1984a). When employees find that their abilities and available resources cannot meet certain job requirements, they feel stressed. However, this stress will not arise because a job is demanding (Karasek, 1979). Therefore, employees identify work stressors after they have cognitively evaluated the task based on their existing coping resources (Lazarus, 1984). Compared with a novice, the pressure felt by people with more work experience is different, even if they are in the same work environment and face the same work tasks. That is to say, experienced employees may feel less or even no pressure, while inexperienced novices may feel overwhelmingly stressed (Robbins and Judge, 2012). When people encounter tasks with requirements that go beyond their abilities, occupational stress will arise based on individual differences (Lazarus and Launier, 1978; Folkman et al., 1987; Ma and Bao, 1999). For example, individuals may be highly motivated to achieve their goals but fail because they do not have enough coping resources or work abilities to complete a given task, or someone may want to avoid hostility from others but not be able to control it themselves. In this case, some individuals will feel stressed due to the loss of control in negative situations, while others will feel no pressure and be indifferent (Kahn and Byosiere, 1992; Avey et al., 2010). In other words, individual differences lead to differences in cognitive evaluations of stress. According to the “stimulus-response” Theory of Stress (Lazarus, 1996), stress is the result of the interaction between environmental stimuli and individual differences (Lazarus and Folkman, 1984b). According to the work stress model proposed by Karasek and Theorell (1990), work stress is the result high psychological needs and low work control. Oenning et al. (2019) have emphasized that psychosocial work stressors are an important type of stress. Among the different psychological factors that influence individual differences, personality characteristics and coping abilities are key components that can induce, relieve, or adjust stress (French and Caplan, 1972; Folkman et al., 1979; Kahn, 1992; Jones et al., 2005; Islam et al., 2022), and work adaptability is such a type coping ability and resource. Individuals with higher levels of work adaptability will experience lower levels of work stress (Yu et al., 2019).

### Work Adaptability

Adaptation is indispensable in work and life (Kumar, 2016). In the context of organizational management, work adaptability is defined as the work experience, habits, and skills that individuals use to adapt to current or changing work tasks and situations (Pulakos et al., 2000; Ployhart and Bliese, 2006). It is not only a specific ability that influences the way individuals adapt to changes and unforeseen work situations (Rottinghaus et al., 2005) but also the psychological ability of individuals to cope with current or anticipated difficulties and changes at work (Kirschenbaum and Daniel, 1986; Skorikov, 2007; Savickas, 2013; Nejad et al., 2021). These changes are related to the job itself, colleagues, or work teams and highlight that adaptability is the most valuable resource for the continuous development of employees (Yu and Zheng, 2013). Kumar (2016) believes that some adaptability qualities, such as an openness to learning new things, the ability to appreciate and communicate with people from different backgrounds, and a comfortableness with uncertainty and change have not only aided his research career but have also helped him prepare to take on leadership roles. Work adaptability is sometimes called occupational adaptability or career adaptability (Nota et al., 2012) and is a type of resource or psychological capital that individuals can develop through interaction, experience, training, and education (Bocciardi et al., 2017). Work adaptability can also help individuals cope with various interpersonal situations and environmental stressors (Paulhus and Martin, 1988; Pulakos et al., 2000; Skorikov, 2007; Nota et al., 2012; Savickas and Porfeli, 2012; Attell et al., 2017). This study thus proposed the following hypothesis:

*Hypothesis 1*: Work stress is negatively related to the work adaptability of young employees. Individuals with higher levels of work adaptability will experience lower levels of work stress.

### Occupational Health

Occupational health is also known as occupational hygiene and is the result of a number of stress factors and mechanism moderators in organizations (Del Pozo-Antúnez et al., 2018). According to an authoritative definition of “occupational health” provided by the Joint Occupational Committee (JOC) of the International Labor Organization and the World Health Organization, occupational health refers to the promotion and maintenance of the best physiological, psychological, and social conditions for employees in various industries. Occupational health involves preventing the health of employees from being affected by work environments, protecting employees from health hazards, and arranging employees in a working environment that is suitable for their physiological and psychological conditions (Xiong, 2019). Lazarus (1984) and Broverman and Lazarus (2006) found that an individual’s psychological and physical wellbeing are more affected by coping strategies than the frequency and intensity of stress. Organizations can prevent unhealthy work behavior by developing their employees’ adaptability (Yu et al., 2019). This study thus proposed the following hypothesis:

*Hypothesis 2*: Work adaptability is positively related to the occupational health of young employees. The higher an individual’s level of work adaptability is, the stronger their occupational health.

### Work Stress and Occupational Health

Work stress always has a great impact on the occupational health of employees. Work stressors cause illness (Jex et al., 1992), anxiety, depression, and exhaustion (Jex, 1998) in employees. Among EU-workers, 25% believe that their health is at risk due to work stress, and this number is even higher for workers in education (42%; Smith et al., 2000; Bardos, 2006). A survey of university teachers’ work stress shows that high work stress and poor physical health are significantly negatively correlated (Dua, 1994). Evidence also shows that work stress causes major health problems, such as cardiovascular diseases (Backé et al., 2012), musculoskeletal disorders (Costa and Vieira, 2010), and periodontitis (Md et al., 2019). Work stress has also been found to increase sick absences (Duijts et al., 2007), lead to poor mental health (Harvey and Edanna, 2017), and reduce positive outcomes at work (Utary, 2014; Svanström, 2016). The amount of work stress directly affects the occupational health levels of employees themselves (Beehr, 2001; Kinman, 2008) and is a significant contributor to increased rates of fatigue, cynicism, and psychological distress (Broberg et al., 2017; Chappelle et al., 2019). Work stress caused by high demand-low control tasks influences the wellbeing of employees (Heyeon et al., 2019; Kern et al., 2020). Work stress from job precautious affects health-related quality of life (Bhattacharya and Ray, 2021), and workplace-related stress can be detrimental to employees’ mental health (Dunnette, 1992; Wan et al., 2020; Bogaerts et al., 2021). Work stress has a long history of influencing the occupational health of employees, and scientific intervention strategies need to be developed in order to combat this issue (Roozeboom et al., 2020).

This study thus proposed the following hypothesis:

*Hypothesis 3*: Work stress is negatively related to the occupational health of young employees. The higher an individual’s level of work stress is, the worse their occupational health becomes.

### The Moderating Role of Work Adaptability in the Relationship Between Work Stressors and Occupational Health

The transactional model of work stress emphasizes the influence that adaptive resources have on occupational health in the dynamic interaction between individuals and the environment (Lazarus et al., 1966; French and Caplan, 1972; Lazarus and Launier, 1978; Lazarus, 1984; Lazarus and Folkman, 1987). Folkman et al. (1987) pointed out that an individual’s adaptive resources play an important role in the face of pressure (Avey et al., 2010). When environmental demands, constraints, and opportunities exceed an individual’s personal reserve of resources, their stress will cause psychological tension, and continuous tension will inevitably affect their physical and mental health (Folkman et al., 1987; Beehr, 1995; David and Carl, 2001). Many researchers have provided supporting evidence of this dynamic from different critical perspectives (Cohen et al., 1983; Baker and Karasek, 2000; David and Carl, 2001; Adamczyk and Segrin, 2015). For instance, French and Caplan (1972) proposed a theoretical model of human adaptation to the environment and claimed that adaptation involves adjusting individual abilities and various possibilities presented at work. If there is no adequate coordination of adaptive resources between personal ability and the environment, then the possibility of anxiety and depression will increase (French and Caplan, 1972; French et al., 1974).

This type of adaptation includes the following two aspects: (1) the ability and resources that an individual has at their disposal when faced with the task of adapting to work-related needs; (2) the supply of environment is provided to meet the adaptation of individual needs. Stress arises when there is a large gap between work needs and the abilities and resources that an individual has at their disposal to meet those needs or between individual needs and the supply resources available in a given environment to meet those needs (Edwards et al., 1998). If an individual believes that they have enough resources or that an organization will provide resources to help them cope with stress and succeed, then they will have strong occupational health and can effectively adapt to stressful situations. Otherwise, individuals fail to adapt, face professional tension, and demonstrate abnormal physiological, psychological, and behavioral symptoms (Lennon et al., 1984; Jex and Bliese, 1999). Both adaptation and maladaptation are the results of the interaction between an individual and their environment, and adaptive resources play an important role in this dynamic (Lofquist and Lloyd, 1984; Li, 1999; Ployhart and Bliese, 2006; Adamczyk and Segrin, 2015). Caplan (1997) have also shown that the characteristics of individuals and their work environment jointly determine the health status of employees (Caplan, 1997). They also found that when the person-environment fit fails, occupational stress will develop (Caplan, 1997). Persistent high levels of long-term stress therefore have an impact on the physiological, psychological, and behavioral health of employees (Li, 1999; Jones et al., 2005).

Hirschi also found that work adaptability is an adaptation resource (Hirschi, 2009; Griffin and Hesketh, 2011). Work adaptability is an important predictor of occupational mental health, and individuals with higher levels of work adaptability can obtain better mental health and achieve more effective career success (Zellars and Perrewe, 2001; Griffin and Hesketh, 2011; Nejad et al., 2021). Work adaptivity as a trait component is positively associated with career satisfaction and career promotability (Tolentino et al., 2014), and some habits of emotional social support appear to protect against burnout (Zellars and Perrewe, 2001). Chen (2009) also found that the work ability of Taiwanese preschool teachers influenced their occupational health and that employees with higher levels of work adaptability experience less job burnout. The research mentioned here was based on the theoretical model of interaction of work stress and the dynamic interaction process among work stress, coping resources, and occupation health. Does this theoretical basis and the relationship between variables apply to young employees at the early stage of career development (Jex and Beer, 1991)? In order to address this question, the present study explored the moderating role that work adaptability plays in the relationship between work stress and occupational stress in young employees as a means of collecting empirical data about the management of work stress in various organizations. Accordingly, the following hypothesis was proposed as:

*Hypothesis 4*: Work adaptability plays a moderating role in the relationship between young employee’s work stress and occupational health.

**Figure 1:**
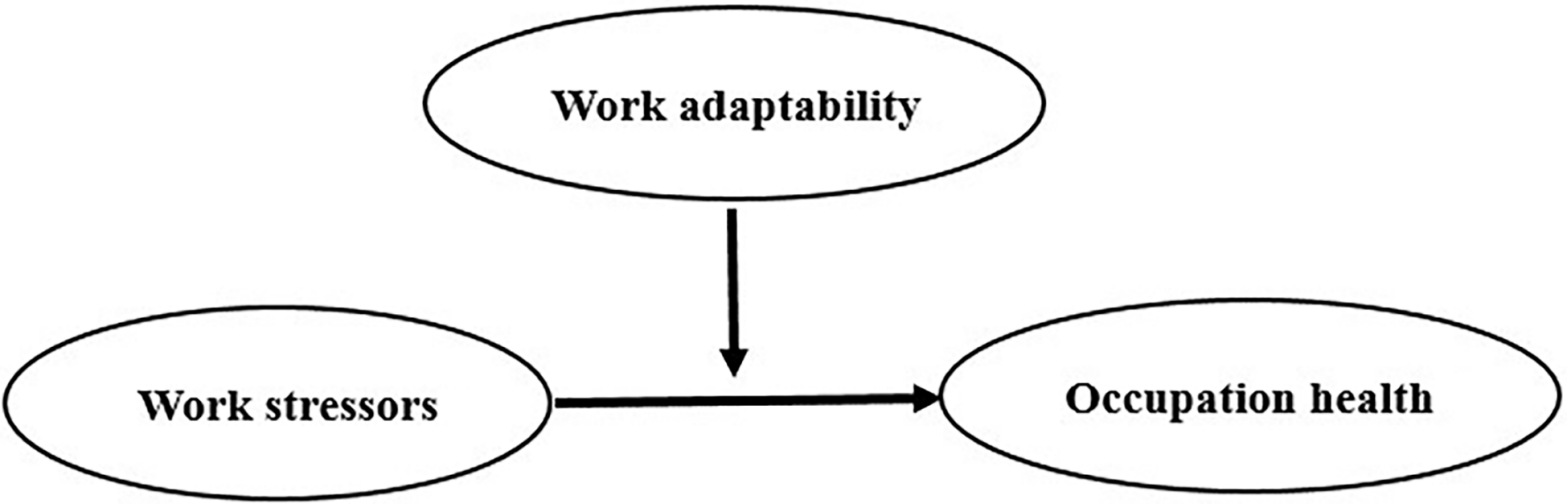
Hypothesized research model.

The proposed integrated model is presented in Figure 1.

## Methodology

### Research Design and Methods

#### Interview Research Stage

In this study, we interviewed 10 on-the-job young employees aged 20–30 years with 2–5 years of work experience who were in the career exploration phase. In terms of age distribution, four people were 20–23 years old, four were 24–27 years old, and two were 28–30 years old. After selecting the interviewees, we conducted semi-structured interviews. Some examples of the questions asked during the interview are “What has happened in the organization that makes you feel stressed?,” “Do these things affect your work and rest or make you dislike your work, leaders, colleagues, etc.?,” “What kind of stress would make you want to leave your job?,” and “Can you cope with your stress now?” (Baker and Karasek, 2000; Diraz et al., 2003; Griffin and Hesketh, 2003). Depending on the responses to these questions, the study referred to the items of the work stressor questionnaire created by Li (1999) while inviting young employees to judge which items were consistent with the stress that they faced at work. The matched items were retained as test items in the research stage. Some items were revised after reviewing the results of the interviews and questionnaire responses.

In order to investigate the role of work adaptability, interviewees were asked to evaluate the behaviors that they use to cope with stress in the workplace. Based on these evaluation results, corresponding measurement items were then selected and applied.

#### Questionnaire Research Stage

Using a non-probability judgment sampling technique, a total of 158 full-time young employees were sampled from 20 different organizations in Zhejiang Province, most of which were involved in the manufacturing and food processing industries. The selected organizations all employed at least 200 people. The researchers brought paper questionnaires to these organizations, and managers distributed them to young employees. Each organization was given an average of 5–10 questionnaires depending on its size to ensure the representativeness of the sample and external validity, and all of the questionnaires were returned 3 days later. For example, organizations that employed 200 people were issued 5–6 questionnaires, while organizations that employed 300–500 people were issued 10 questionnaires. Thirty questionnaires were rejected due to incomplete information, leaving 128 valid questionnaires. A power analysis was also conducted with an effect size of 0.15 and a probability error of 0.05, and the results indicated that the sample size was adequate enough to perform analysis for this study. The age distribution of the respondents was between 18 and 29 years old. With respect to educational background, 22 respondents had bachelor’s degrees, 59 had college degrees, and 47 held high school degrees. Forty-four of the respondents were married, and 84 were unmarried. The sample included 57 males and 70 females, and one respondent did not fill in this field.

#### Measures

The questionnaire used in this study consisted of four sections: (a) demographic information (gender, age, and education), (b) the work stressor scale, (c) the work adaptability scale, and (d) the occupational health scale.

##### Work Stressor

Work stressors are rated based on the work stressors scale, which was revised and established based on the interview survey of young employees and the work stressors questionnaire compiled by Li (1999). The scale has 26 items and is divided into seven dimensions: lack of achievement development stress (*a* = 0.736), unhealthy organization atmosphere stress (*a* = 0.812), highly difficult task stress (*a* = 678), poor working conditions stress (*a* = 0.731), interpersonal relationship stress (*a* = 0.825), role conflict stress (*a* = 0.882), and lack of work meaning stress (*a* = 0.758). Respondents were asked to use a five-point Likert scale ranging from 1 (strongly disagree) to 5 (strongly agree) to express the extent to which they agreed with each item. Higher scores indicate that a respondent faces strong work stressors.

##### Work Adaptability

The items used for the work adaptability scale come from the work adaptability description questionnaire compiled by Pulakos et al. (2000), the semi-structures interviews, and the results of the preliminary questionnaires. The work adaptability description questionnaire was originally developed in English and then translated into Chinese for use in this study. In order to improve the accuracy of the translations, a back-translation method was adopted (Brislin, 1980). A translator translated the original English scale into Chinese, and then, a second translator translated the scale back into English. Finally, a third translator combined the original, translated, and back-translated scales. The three versions of the scale were then compared, and the translation was revised to ensure the equivalence of the questionnaire content. Then, based on the interview research, the final questionnaire measurement items were determined. The scale has 31 items divided into four dimensions: complex and innovative problem-solving adaptability, learning adaptability, environmental adaptability, and physical condition adaptability. Respondents used a seven-point Likert scale ranging from 1 (completely disagree) to 7 (completely agree) to express the extent to which they agreed with each item. Higher scores indicated a stronger level of work adaptability. In this study, Cronbach’s alpha is 0.931.

##### Occupation Health

This study used the occupational health scale to evaluate the negative emotions and psychological and physical conditions of young employees (House and Rizzo, 1972; Ma and Liang, 1997). The full scale consisted of 11 items, such as “I feel depressed because of my troubles at work” and “I often feel tired after work.” Respondents used a five-point Likert scale ranging from 1 (strongly disagree) to 5 (strongly agree) to express the extent to which they agreed with each item. Higher scores indicated lower levels of occupational health. In this study, Cronbach’s alpha is 0.780.

##### Statistical Methods

SPSS 20.0 was used to perform data analysis. The main objective of the statistical process used in this study was to analyze the moderating effect of work adaptability in the relationship between work stress and occupational health.

## Results

### Descriptive Statistics and Correlations

The maximum value, minimum value, means, standard deviations, and reliabilities among the study variables are presented in Supplementary Table 1. As shown in Supplementary Table 1, the average for stress related to poor working conditions is the lowest, which indicates that the respondents felt little to no stress in this category, while the total work stress score and scores of work stressors from various dimensions fell below the mid-level. The average of work adaptability is between the general and the somewhat consistent level, and its overall score is in the upper middle level, reflecting that the work adaptability of the young group has not reached the level of skilled workers, but is between the level of novice and semi-skilled workers, and only a few of them have achieved skilled level. In terms of occupational health scores, the average for the negative emotions and psychological and physical conditions that young employees face is between occasional and sometimes occurrence level, and the overall level for occupational health was above average. The internal consistency reliability coefficients of the scales used in this study are between 0.69 and 0.95, which meets the requirements of psychological measurement.

The correlation matrix is reported in Supplementary Table 2. As shown in Supplementary Table 2, work adaptability is significantly correlated with poor working condition stress, interpersonal relationship stress, role conflict stress, and work stressor score (*p* < 0.01), and it is also remarkably associated with lack of achievement development stress (*p* < 0.05). Occupational health scores are significantly correlated with lack of achievement development stress, unhealthy organization atmosphere stress, highly difficult task stress, work stressor score, and work adaptability (*p* < 0.01), but they are not significantly correlated with interpersonal relationship stress, role conflict stress, poor working condition stress, and lack of work meaning stress.

### Hypothesis Testing

#### Common Method Bias

A Harman single-factor test (Podsakoff and Organ, 1986) was conducted, and it was found that the explained variance of the first principal component was 24.68%. Because this result is below the cut-off value of 50% (Podsakoff et al., 2003), we concluded that common method bias was not a serious problem.

#### Moderation Analysis

Moderator variables are variables that can affect the intensity and direction of the relationship between predictor variables and criterion variables. A moderator variable always acts as an independent variable and indicates when a certain effect exists (Baron and Kenny, 1986).

**Figure 2:**
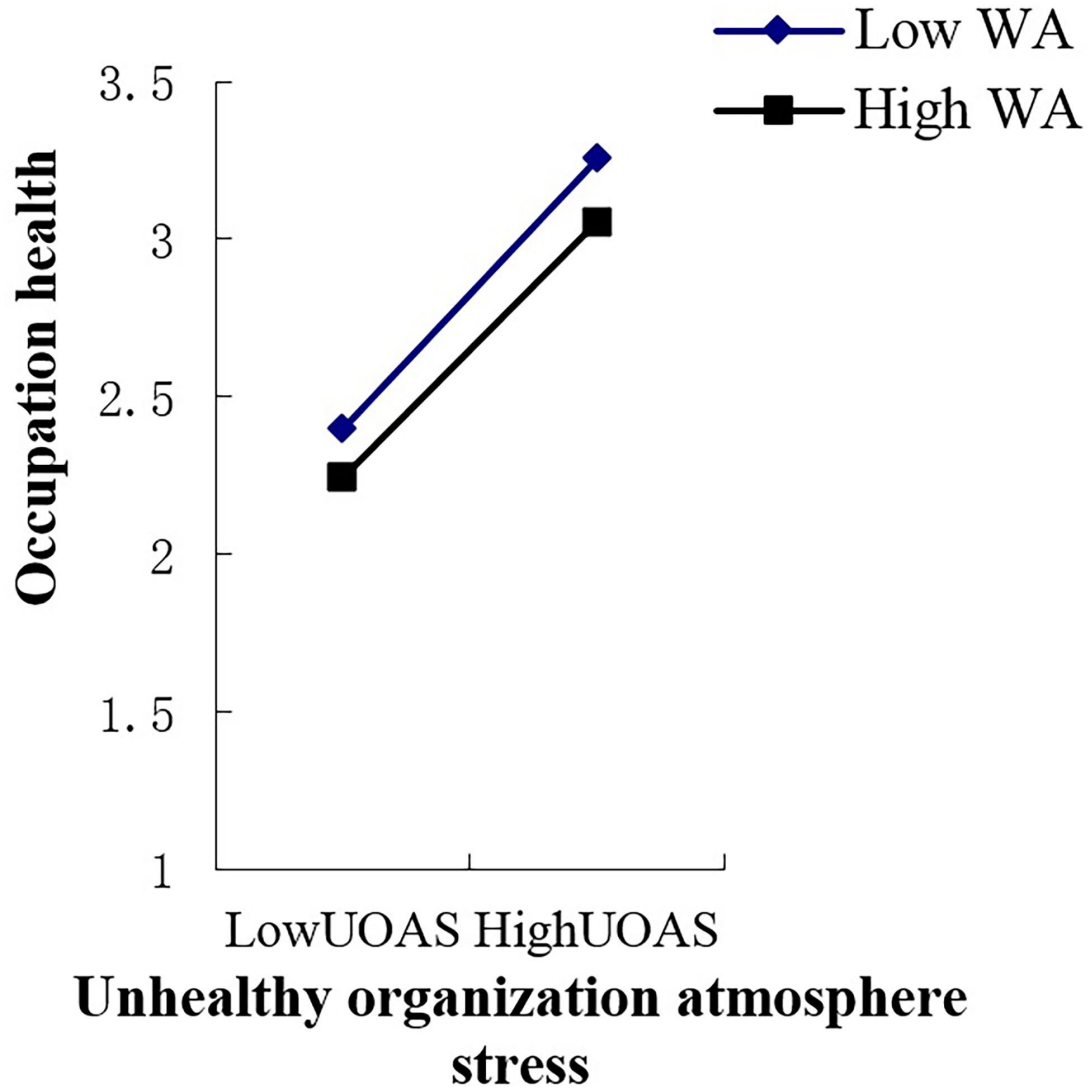
Interactive effects of WA and UOAS on OH. WA, work adaptability; UOAS, unhealthy organization atmosphere stress; OH, occupation health.

**Figure 3:**
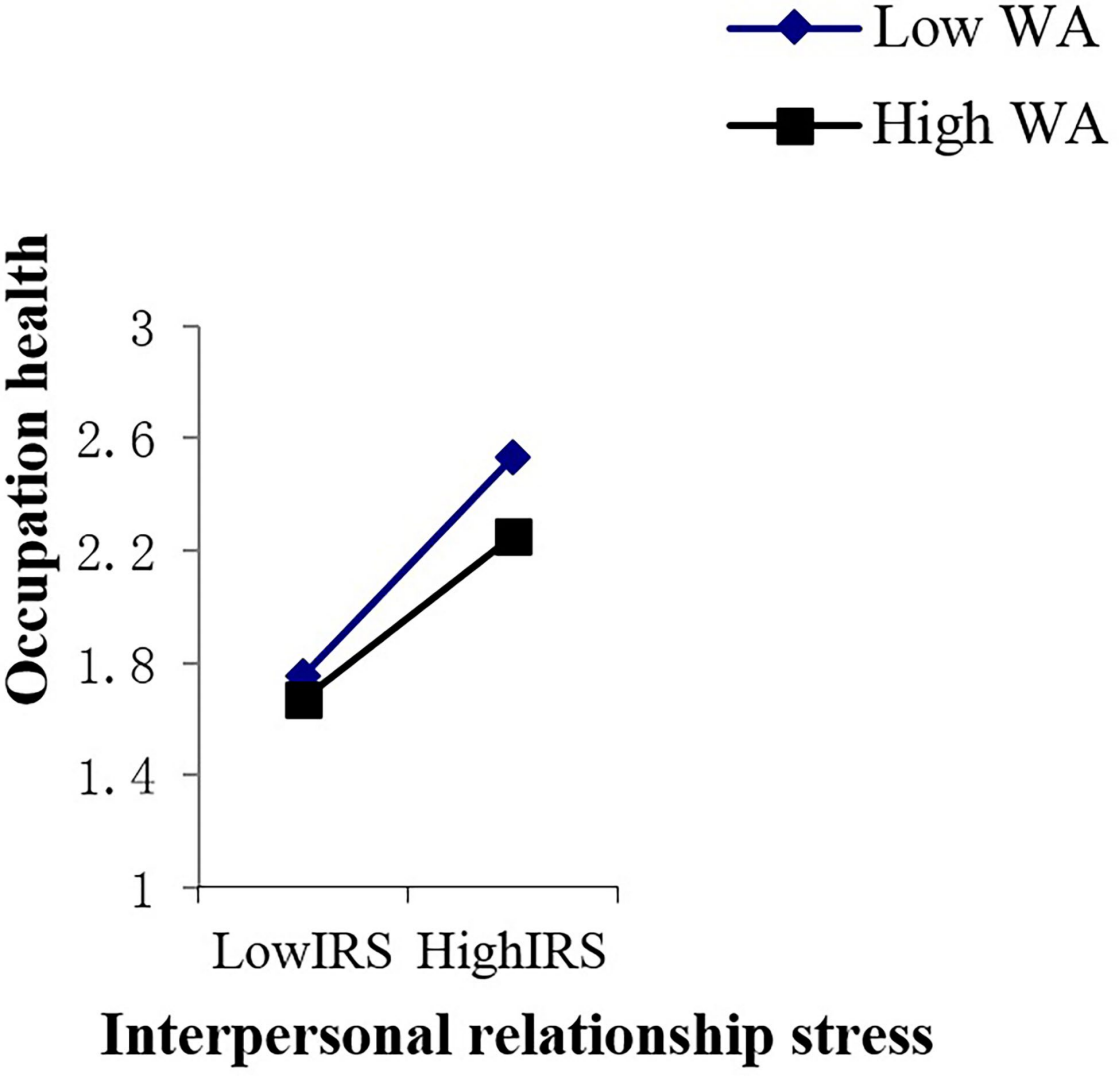
Interactive effects of WA and IRS on OH. WA, work adaptability; IRS, interpersonal relationship stress; OH, occupation health.

**Figure 4:**
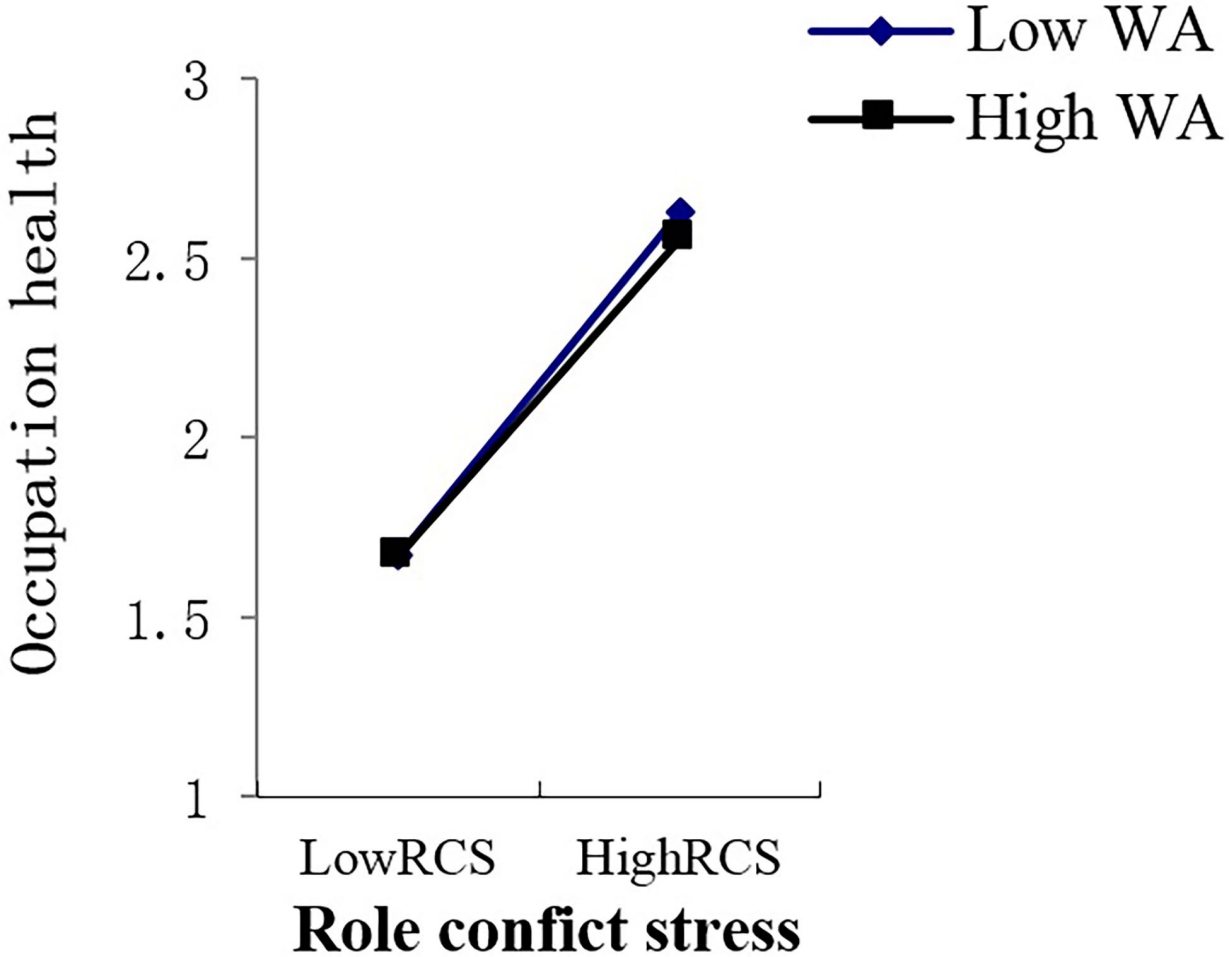
Interactive effects of WA and RCS on OH. WA, work adaptability; RCS, role conflict stress; OH, occupation health.

**Figure 5:**
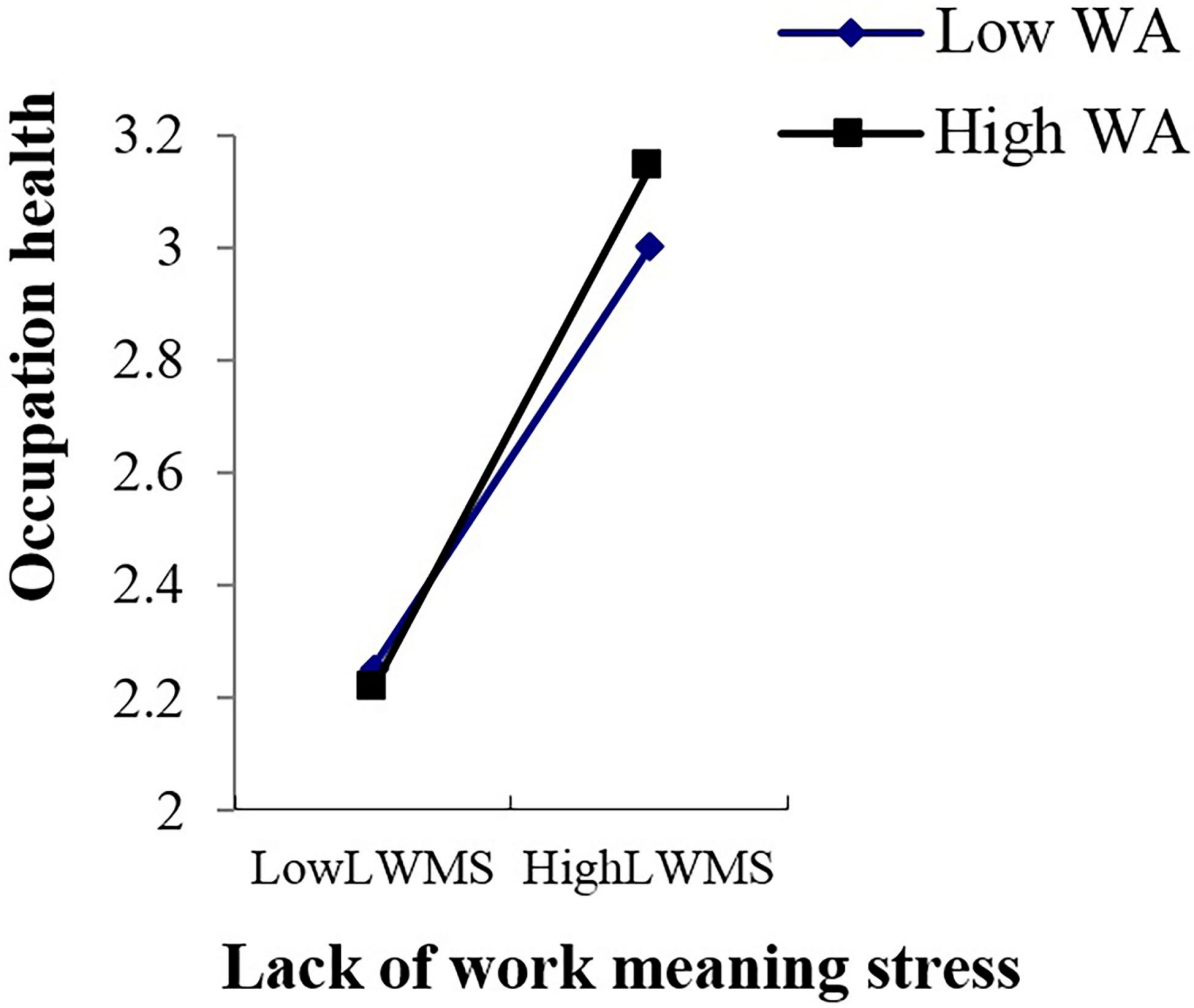
Interactive effects of WA and LWMS on OH. WA, work adaptability; LWMS, lack of work meaning stress; OH, occupation health.

**Figure 6:**
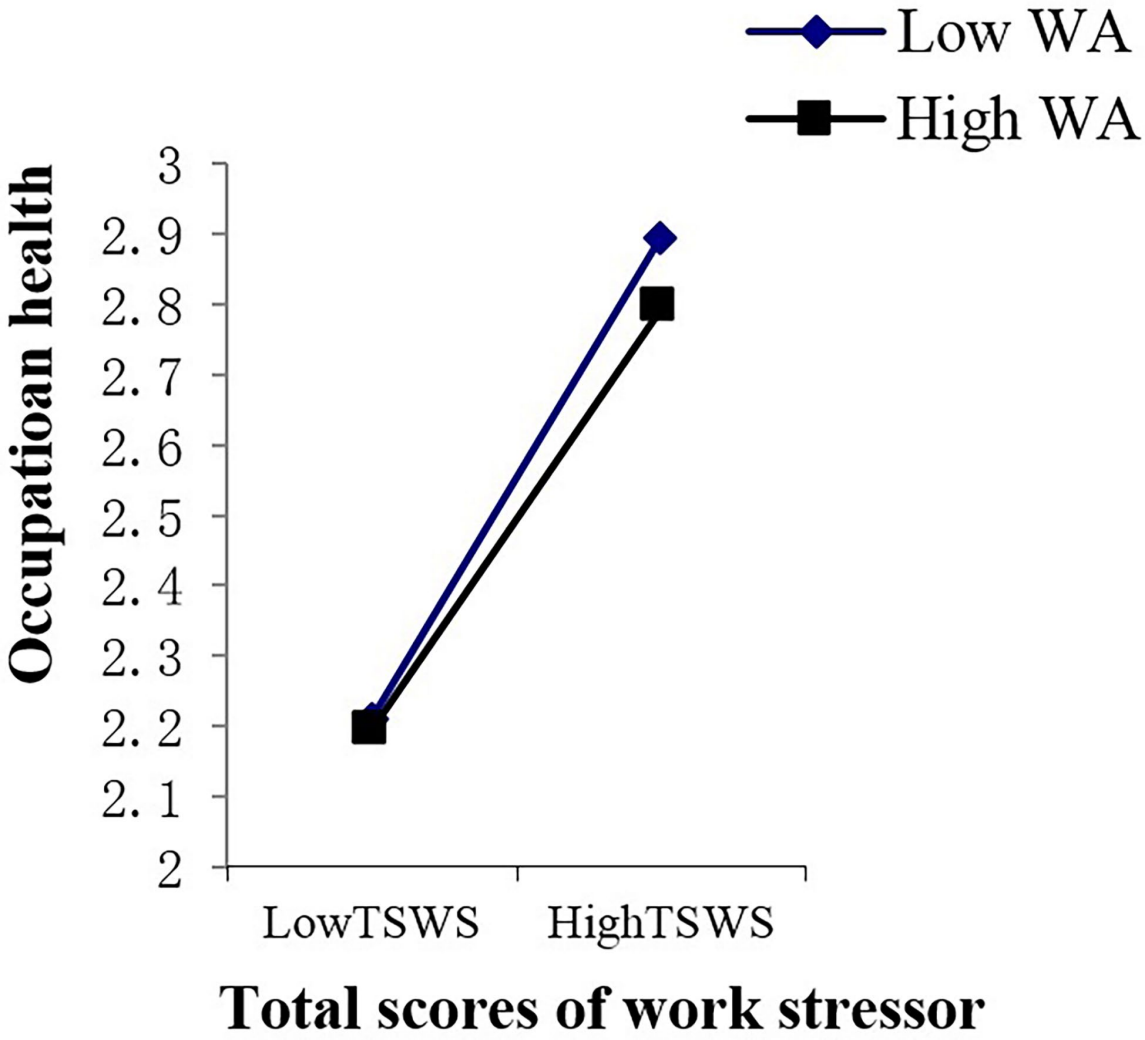
Interactive effects of WA and TSWS on OH. WA, work adaptability; TSWS, total scores of work stress; OH, occupation health.

In this study, work adaptability was used as the independent variable, and the effect of the work stressors’ total score and various dimensional factors of work stress on occupational health were used as dependent variables to estimate the line relationship that exists between them. In the end, an obvious line relationship was found (Baron and Kenny, 1986; Saks, 1995). Therefore, the multiple linear regression of “enter option” was adopted to analyze the moderator role. Collectively, this study included eight analytical aspects of work stressors, one moderator variable, and one dependent variable. Thus, there were a total of eight groups used to analyze the moderator role, that is [8 (work stressor) × 1 (work adaptability) × 1 (occupation health)]. As shown in Supplementary Table 3, the main effect of young employees’ work adaptability on occupational health is significant in the various dimensions of work stress; the moderating mechanism of young employees’ work adaptability exists in the relationships between lack of work meaning stress and occupational health (*p* < 0.05), role conflict stress and occupation health (*p* < 0.01), interpersonal relationship stress and occupational health (*p* < 0.05), unhealthy organization atmosphere stress and occupation health (*p* < 0.05), and work stressor score and occupational health (*p* < 0.05). These moderating effects are depicted in Figures 2–6. Although lack of achievement development stress, highly difficult task stress, and poor working condition stress can predict occupational health (*p* < 0.01), the interaction effect between these variables and work adaptability in the prediction of occupational health is not significant. We thus believe that no moderating effects are present in this dynamic.

The moderating role is illustrated as in Figures 2–6.

## Discussion

From the perspective of work stress interaction theory, this study highlights the relationship that exists between the work stress, work adaptability, and occupational health of young employees who are at the stage of career exploration. Empirical evidence reveals that a high level of work adaptability helps young employees at the career exploration stage cope with work stress and maintain occupational health. Young employees with high work adaptability have better occupational health status when facing stressful events than those with low work adaptability. Young employees with high work adaptability can use their own coping resources or integrate the coping resources in the environment to actively adapt and effectively deal with difficult problems at work, which not only reduces the level of pressure perception of stress events but also leads to lower self-perceived occupational health threats. It also allows employees to develop and improve their work adaptability. In the present study, this type of moderating effect emerges between role conflict stress and occupational health, interpersonal relationship stress and occupational health, unhealthy organization atmosphere stress and occupational health, and total work stressor scores and occupational health. In order to cope with these types of work pressures and maintain occupational health, young employees need to achieve a higher level of work adaptability.

This study also found that when young employees with high job adaptability faced the pressure of lack of job meaning during the career exploration stage, their occupational health status was worse than those young employees with low work adaptability. This means that the moderating role in the relationship between lack of work meaningful stress and occupational health is complex. It might be the case that although young employees with high work adaptability have the resources and ability necessary to adapt to a high level of lack of work meaning stress, they feel that this kind of adaptation is meaningless and painful and are reluctant to deal with that stress. The above conclusion indicates that for young employees, the higher their work adaptability is, the more they need to engage in jobs that can have a significant impact on the work and life of others in and out of an organization.

With regard to hypotheses and statistical verification, the correlation analysis results in Supplementary Table 2 indicate that the correlations between lack of work meaning stress and occupation health, role conflict stress and occupation health, and interpersonal relationship stress and occupation health were not significant. It seems that these factors of work stress have nothing to do with the occupational health of young employees. However, with the introduction of the moderator variable (namely, work adaptability), the impact of lack of work meaning stress, role conflict stress, and interpersonal relationship stress on occupational health becomes clear. The results of the correlation analysis and the multiple linear regression analysis have confirmed the hypothesis that work adaptability plays a moderating role in the relationship between lack of work meaning stress, role conflict stress, interpersonal relationship stress, and occupation health (Baron and Kenny, 1986).

In conclusion, it is worthwhile for organizations to work on continuously improving the work adaptability of their young employees in order to help mitigate work stressors and promote occupational health (Jones et al., 2005).

### Theoretical Contribution

By constructing a work stress management model with work adaptability as a moderating variable and conducting empirical research, this study verified, enriched, and developed the work stress management interaction theory model. In the literature on HRM and stress management, research that treats work adaptability as the moderating variable is limited. This study reveals the positive role that work adaptability plays in stress management for young employees, especially with respect to combating stress and maintaining occupational health. This research also highlights the direction that young employees should take in order to develop their potential and actively respond to various types of stressful events that can take place during the career exploration stage. More specifically, this study highlights that work adaptability can help young employees in the early career exploration stage cope with work stress and maintain occupational health. In the actual work environment, under the premise that individual work values do not deviate from the direction of organizational values, it is of great importance to plan and develop employees’ work adaptability continuously and in the correct way. Doing so can help optimize employees’ career planning and lead to the healthy development of both the organization and its individual workers.

The moderating effect of work adaptability suggests that occupational health effects that stem from interactions between work adaptability and work stress occurred between the total work adaptability level and a certain dimension of work stress. In order to effectively deal with the pressure of a certain dimension, the total score of work adaptability must be used to deal with this situation. In this study, unhealthy organization atmosphere stress, interpersonal relationship stress, role conflict stress, and work stressor score belong to this situation. This means that people with high work adaptability have better occupational health in the face of these types of pressures than people with low work adaptability. This shows that work adaptability has a positive effect on young employees’ occupational health and ability to cope with stress. These results not only provide theoretical and empirical data support and a reference basis for follow-up research in related fields but also enrich and develop the pressure management theory in the context of young employees who are at the career exploration stage.

### Practical Implications

According to the interaction theory of work stress management, stress is the result of personal cognitive evaluation in the process of dynamic interaction among organizations, employees, and stressful jobs (Folkman et al., 1987; David and Carl, 2001; Beehr, 2014). Therefore, work stress should be dealt with from the perspectives of organizational and individual management to avoid negative stress and occupationally unhealthy factors that may emerge in the process of interaction between all parties (Jones et al., 2005; Kim et al., 2014). The outcomes of this research offer several practical implications for the managers and young employees of organizations operating in Chinese manufacturing and food processing industries. First, during the career exploration stage, work adaptability helps young employees cope with stress and maintain occupational health. Organizations can thus assess a candidate’s level of work adaptability at the recruitment stage to ensure that they can meet the requirements of certain job tasks. Moreover, if the cost of short-term recruitment and employment is considered, the organization can make plans to develop training that continuously fosters work adaptability in new employees at various stages, incorporate the cultivation of work adaptability into the career planning of employees, and to use the strategy of employing, retaining, and cultivating management ideas that consolidate the growth path of employees’ work adaptability. Organizations not only need to train young people on the knowledge and theory of work adaptability but also cultivate their skills and practical experience in this area. Only in this way can work adaptability be used as an effective response resource that can be truly applied to the practice of work stress management.

Secondly, organizations need to design a career development path with a sense of accomplishment for employees, enable their staff to develop stronger work adaptability and motivations, encourage them to move toward more attractive goals, and help them mitigate stress and make efforts to move toward the top of their desired career. Thirdly, organizations should also focus on providing better working conditions and meaningful jobs for young employees, reducing role conflicts, and creating a sound organizational atmosphere. The management of young employees’ work stress is a systematic project, and a supportive organizational culture and atmosphere are beneficial for both young employees and organizations. It can give young employees a long-standing sense of belonging within the organization, have a positive impact on work stress, and ensure the long-term and sustainable development of the two parties. The management method for organizations and individuals to actively adapt to work stress, and the countermeasure of integrating various resources of employees and organizations for active adaptation are also a good key to break through the dilemma of the work stress interaction theoretical model. Organizations that are in a position to do so can incorporate the improvement of employees’ work adaptability into the organization’s performance allocation scheme or recognition awards, which can help create an environment conducive to the development of employees’ work adaptability from the perspective of organizational compensation management.

From the perspective of self-development, the research findings also suggest that young employees should correct their practical attitude in order to manage work stress effectively. The development of work adaptability is a long process (Munz et al., 2001) Therefore, young employees should focus on the present in their actual work. They should first continue to improve their work adaptability and learn to evaluate and cope with their stress in a rational way (Hirschi, 2009). Based on their existing level of work adaptability, young employees should do their best to improve their work adaptability and complete the tasks that are assigned to them. For tasks that require employees to reach beyond their coping resources, workers could learn to reduce their cognitive sense of stress and seek out social support if possible (Maslach and Leiter, 1997). For tasks that cannot be completed, employees can tell their supervisors the truth and ask them to assign the task to someone who has more experience in order to prevent the work from being delayed and avoid continuous high stress. In order to address lack of work meaning stress, it is important to recognize that young employees who are unsophisticated or inexperienced tend to view what they do as meaningless and are eager to tackle work that is beyond their current abilities. Therefore, when judging whether certain tasks are significant or not, employees should seek out people with rich work experience for guidance. At that point, young employees can conduct stress cognitive evaluation. In this way, employees can reduce unnecessary stress, adapt more easily to their work, and achieve steady, sustainable, and effective development.

Second, in the context of stress management, young employees should learn to adopt different coping strategies when facing different types of stressors. Some stressors, such as highly difficult task stress, can be dealt with through low-stress sense evaluation strategies. However, lack of work meaning stress and other stressors should be coped with using the method of pressure release. Furthermore, in order to mitigate their stress, young employees should learn how to use multiple coping methods, such as attention transfer, emotional relaxation, and seeking social support. When facing interpersonal relationship stress, employees should adopt the above methods (low stress sensed evaluation, stress release, response resource accumulation and training, and seeking social support) based on the actual complexity and difficulty of the situation. In other words, young employees should not only learn to mitigate stress by evaluating the source of various stressors but also adopt countermeasures and develop intelligent stress management in order to promote their occupational health. Young employees must understand that even if work adaptability cannot be improved in the short term, they can still find various ways to deal with work stress and foster occupational health.

## Limitations and Future Research

This study explored the work adaptability-based management of young employees’ work stress by means of interviews and questionnaires. It provides empirical support for the management of young employees’ work stress and reveals the role that work adaptability plays in the management of work stress and promotion of occupational health in young employees. This study also uncovers the role of work adaptability as a moderator in the association between work stress and occupational health at the career exploration stage. Outcomes of this study confirm that high levels of work adaptability can help young employees deal with work stress and maintain occupational health. In addition, this study shows that work adaptability plays a moderating role in the relationship between work stress and occupational health in the context of young employees who work in the Chinese manufacturing and food processing industries. The current study paves the way for future researchers and can help guide stress management practitioners in China and similar cultural contexts as they implement effective strategies designed to help manage work stress and promote occupational health and work adaptability in their organizations.

It is also important to mention that this study does have several limitations. Data and results based on cross-sectional research cannot provide comprehensive theoretical support for field practices, so future studies should investigate this issue from different perspectives to draw more comprehensive and sufficient conclusions. For instance, researchers could use the questionnaire method of quantitative research to collect longitudinal research data, conduct a case tracking study by conducting quantitative and qualitative research, and design a longitudinal training intervention experiment based on the improvement of work adaptability. Going forward, we will utilize a variety of research designs to demonstrate the research conception from multiple perspectives. In this way, we can provide more comprehensive, reliable, and effective reference materials and practical guidance for the management of young employees’ work stress.

## Data Availability Statement

The raw data supporting the conclusions of this article will be made available by the authors, without undue reservation.

## Ethics Statement

The studies involving human participants were reviewed and approved by Ethics Committee of Hangzhou Normal University. Written informed consent for participation was not required for this study in accordance with the national legislation and the institutional requirements.

## Author Contributions

HZ and QZ designed the study and revised the manuscript. HZ collected and analyzed the data and draft the manuscript. All the authors contributed to the article and approved the submitted version.

## Funding

This research was funded by the second batch of teaching reform research projects in Zhejiang’s higher education sector during the 13th Five-Year Plan: the exploration and practice of “three questions and three explorations” teaching mode of “Students’ Mental Health Education” based on the Spoc flip classroom (no. jg20190378).

## Conflict of Interest

The authors declare that the research was conducted in the absence of any commercial or financial relationships that could be construed as a potential conflict of interest.

## Publisher’s Note

All claims expressed in this article are solely those of the authors and do not necessarily represent those of their affiliated organizations, or those of the publisher, the editors and the reviewers. Any product that may be evaluated in this article, or claim that may be made by its manufacturer, is not guaranteed or endorsed by the publisher.
