# Supplementary figures and images for: Work Stressors and Occupational Health of Young Employees: The Moderating Role of Work Adaptability
Source: Front Psychol. 2022 Apr 26;13:796710. doi: 10.3389/fpsyg.2022.796710 (PMC9088676; doi:10.3389/fpsyg.2022.796710)

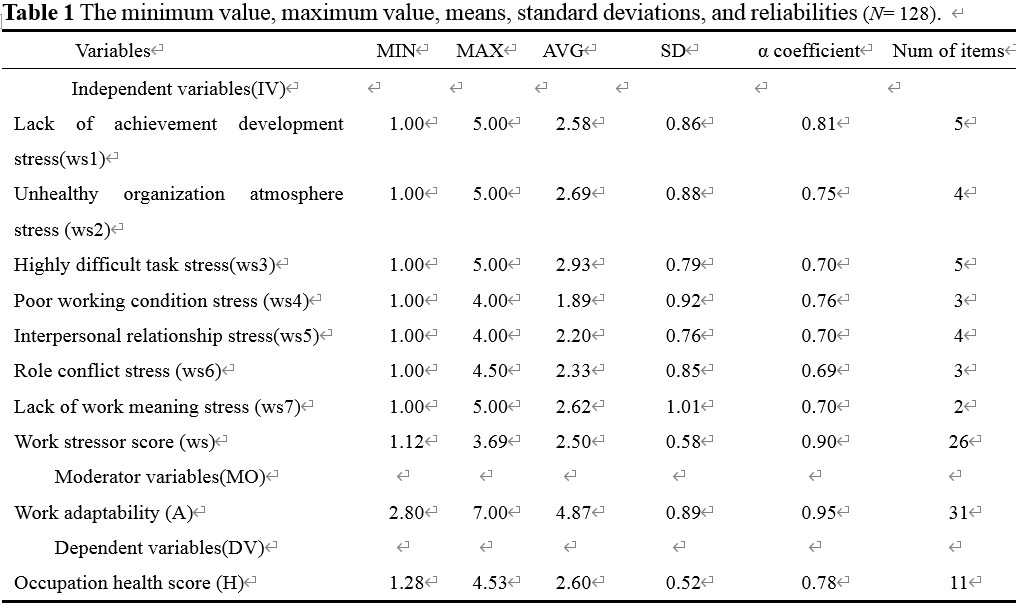

Supplement: Supplementary file 1 [file Image_1.jpeg]

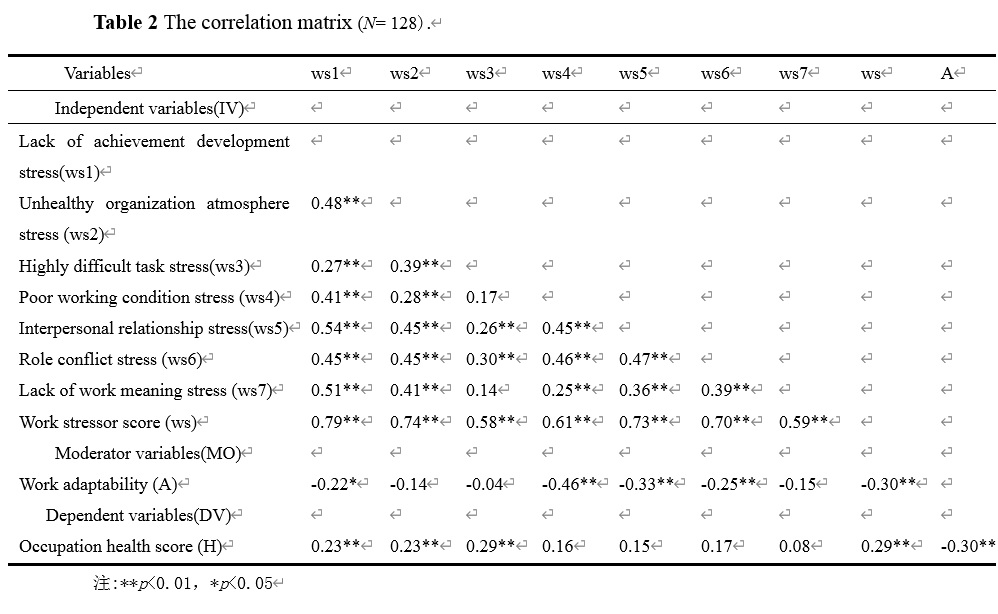

Supplement: Supplementary file 2 [file Image_2.jpeg]

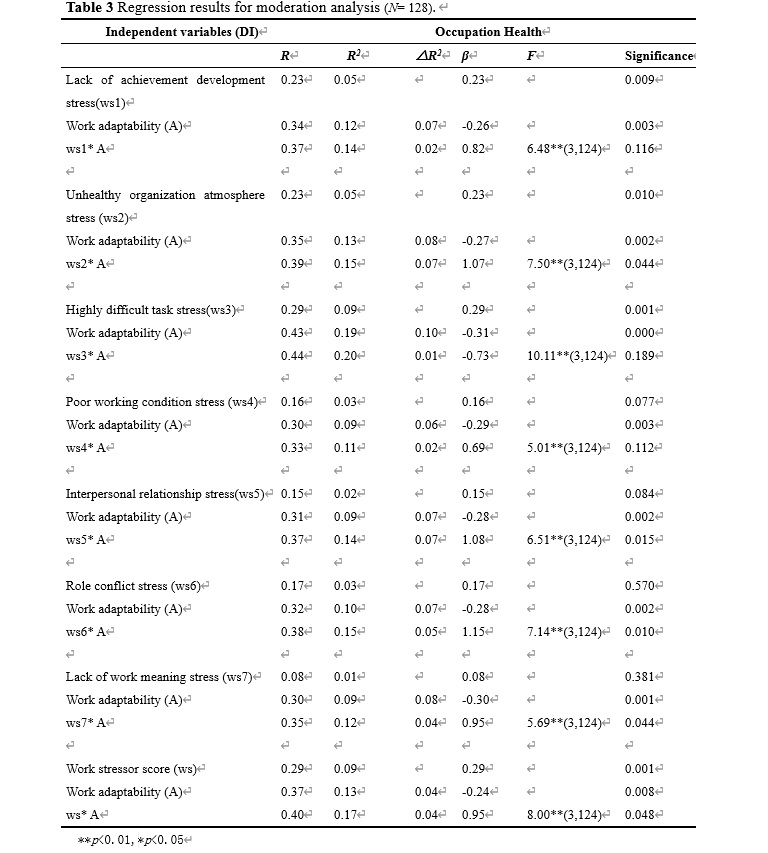

Supplement: Supplementary file 3 [file Image_3.jpeg]
