# Supplementary material for: Work Stressors and Occupational Health of Young Employees: The Moderating Role of Work Adaptability
Source: Front Psychol. 2022 Apr 26;13:796710. doi: 10.3389/fpsyg.2022.796710 (PMC9088676; doi:10.3389/fpsyg.2022.796710)
Supplement: Supplementary file 4 [file Table_1.docx]

**Table 1** The minimum value, maximum value, means, standard deviations, and reliabilities (*N*=128).

| Variables | MIN | MAX | AVG | SD | α coefficient | Num of items |
| --- | --- | --- | --- | --- | --- | --- |
| Independent variables(IV) |  |  |  |  |  |  |
| Lack of achievement development stress(ws1) | 1.00 | 5.00 | 2.58 | 0.86 | 0.81 | 5 |
| Unhealthy organization atmosphere stress (ws2) | 1.00 | 5.00 | 2.69 | 0.88 | 0.75 | 4 |
| Highly difficult task stress(ws3) | 1.00 | 5.00 | 2.93 | 0.79 | 0.70 | 5 |
| Poor working condition stress (ws4) | 1.00 | 4.00 | 1.89 | 0.92 | 0.76 | 3 |
| Interpersonal relationship stress(ws5) | 1.00 | 4.00 | 2.20 | 0.76 | 0.70 | 4 |
| Role conflict stress (ws6) | 1.00 | 4.50 | 2.33 | 0.85 | 0.69 | 3 |
| Lack of work meaning stress (ws7) | 1.00 | 5.00 | 2.62 | 1.01 | 0.70 | 2 |
| Work stressor score (ws) | 1.12 | 3.69 | 2.50 | 0.58 | 0.90 | 26 |
| Moderator variables(MO) |  |  |  |  |  |  |
| Work adaptability (A) | 2.80 | 7.00 | 4.87 | 0.89 | 0.95 | 31 |
| Dependent variables(DV) |  |  |  |  |  |  |
| Occupation health score (H) | 1.28 | 4.53 | 2.60 | 0.52 | 0.78 | 11 |
