# Supplementary material for: Work Stressors and Occupational Health of Young Employees: The Moderating Role of Work Adaptability
Source: Front Psychol. 2022 Apr 26;13:796710. doi: 10.3389/fpsyg.2022.796710 (PMC9088676; doi:10.3389/fpsyg.2022.796710)
Supplement: Supplementary file 5 [file Table_2.docx]

**Table 2** The correlation matrix (*N*= 128).

| Variables | ws1 | ws2 | ws3 | ws4 | ws5 | ws6 | ws7 | ws | A |
| --- | --- | --- | --- | --- | --- | --- | --- | --- | --- |
| Independent variables(IV) |  |  |  |  |  |  |  |  |  |
| Lack of achievement development stress(ws1) |  |  |  |  |  |  |  |  |  |
| Unhealthy organization atmosphere stress (ws2) | 0.48** |  |  |  |  |  |  |  |  |
| Highly difficult task stress(ws3) | 0.27** | 0.39** |  |  |  |  |  |  |  |
| Poor working condition stress (ws4) | 0.41** | 0.28** | 0.17 |  |  |  |  |  |  |
| Interpersonal relationship stress(ws5) | 0.54** | 0.45** | 0.26** | 0.45** |  |  |  |  |  |
| Role conflict stress (ws6) | 0.45** | 0.45** | 0.30** | 0.46** | 0.47** |  |  |  |  |
| Lack of work meaning stress (ws7) | 0.51** | 0.41** | 0.14 | 0.25** | 0.36** | 0.39** |  |  |  |
| Work stressor score (ws) | 0.79** | 0.74** | 0.58** | 0.61** | 0.73** | 0.70** | 0.59** |  |  |
| Moderator variables(MO) |  |  |  |  |  |  |  |  |  |
| Work adaptability (A) | -0.22* | -0.14 | -0.04 | -0.46** | -0.33** | -0.25** | -0.15 | -0.30** |  |
| Dependent variables(DV) |  |  |  |  |  |  |  |  |  |
| Occupation health score (H) | 0.23** | 0.23** | 0.29** | 0.16 | 0.15 | 0.17 | 0.08 | 0.29** | -0.30** |

***p*<0.01，**p*<0.05
