# Supplementary material for: Work Stressors and Occupational Health of Young Employees: The Moderating Role of Work Adaptability
Source: Front Psychol. 2022 Apr 26;13:796710. doi: 10.3389/fpsyg.2022.796710 (PMC9088676; doi:10.3389/fpsyg.2022.796710)
Supplement: Supplementary file 6 [file Table_3.docx]

**Table 3** Regression results for moderation analysis (*N*= 128).

| **Independent variables (DI)** | **Occupation Health** | | | | | |
| --- | --- | --- | --- | --- | --- | --- |
|  | ***R*** | ***R^2^*** | ***△R^2^*** | ***β*** | ***F*** | **Significance** |
| Lack of achievement development stress(ws1) | 0.23 | 0.05 |  | 0.23 |  | 0.009 |
| Work adaptability (A) | 0.34 | 0.12 | 0.07 | -0.26 |  | 0.003 |
| ws1* A | 0.37 | 0.14 | 0.02 | 0.82 | 6.48**(3,124) | 0.116 |
|  |  |  |  |  |  |  |
| Unhealthy organization atmosphere stress (ws2) | 0.23 | 0.05 |  | 0.23 |  | 0.010 |
| Work adaptability (A) | 0.35 | 0.13 | 0.08 | -0.27 |  | 0.002 |
| ws2* A | 0.39 | 0.15 | 0.07 | 1.07 | 7.50**(3,124) | 0.044 |
|  |  |  |  |  |  |  |
| Highly difficult task stress(ws3) | 0.29 | 0.09 |  | 0.29 |  | 0.001 |
| Work adaptability (A) | 0.43 | 0.19 | 0.10 | -0.31 |  | 0.000 |
| ws3* A | 0.44 | 0.20 | 0.01 | -0.73 | 10.11**(3,124) | 0.189 |
|  |  |  |  |  |  |  |
| Poor working condition stress (ws4) | 0.16 | 0.03 |  | 0.16 |  | 0.077 |
| Work adaptability (A) | 0.30 | 0.09 | 0.06 | -0.29 |  | 0.003 |
| ws4* A | 0.33 | 0.11 | 0.02 | 0.69 | 5.01**(3,124) | 0.112 |
|  |  |  |  |  |  |  |
| Interpersonal relationship stress(ws5) | 0.15 | 0.02 |  | 0.15 |  | 0.084 |
| Work adaptability (A) | 0.31 | 0.09 | 0.07 | -0.28 |  | 0.002 |
| ws5* A | 0.37 | 0.14 | 0.07 | 1.08 | 6.51**(3,124) | 0.015 |
|  |  |  |  |  |  |  |
| Role conflict stress (ws6) | 0.17 | 0.03 |  | 0.17 |  | 0.570 |
| Work adaptability (A) | 0.32 | 0.10 | 0.07 | -0.28 |  | 0.002 |
| ws6* A | 0.38 | 0.15 | 0.05 | 1.15 | 7.14**(3,124) | 0.010 |
|  |  |  |  |  |  |  |
| Lack of work meaning stress (ws7) | 0.08 | 0.01 |  | 0.08 |  | 0.381 |
| Work adaptability (A) | 0.30 | 0.09 | 0.08 | -0.30 |  | 0.001 |
| ws7* A | 0.35 | 0.12 | 0.04 | 0.95 | 5.69**(3,124) | 0.044 |
|  |  |  |  |  |  |  |
| Work stressor score (ws) | 0.29 | 0.09 |  | 0.29 |  | 0.001 |
| Work adaptability (A) | 0.37 | 0.13 | 0.04 | -0.24 |  | 0.008 |
| ws* A | 0.40 | 0.17 | 0.04 | 0.95 | 8.00**(3,124) | 0.048 |

***p*<0.01, **p*<0.05
