# Supplementary material for: Work Stressors and Occupational Health of Young Employees: The Moderating Role of Work Adaptability
Source: Front Psychol. 2022 Apr 26;13:796710. doi: 10.3389/fpsyg.2022.796710 (PMC9088676; doi:10.3389/fpsyg.2022.796710)
Supplement: Supplementary file 7 [file Table_4.docx]

|  | **Work Stressors** |
| --- | --- |
| 1. | The structure of my work is complex and difficult. |
| 2. | The amount of work that the unit gave me was very inappropriate. |
| 3. | My job is monotonous and lacks variety. |
| 4. | The working hours are demanding and the pace is too fast. |
| 5. | My working environment and facilities are rather difficult. |
| 6. | My working conditions are not good. |
| 7. | The group atmosphere of the unit makes me feel depressed. |
| 8. | I often don't know what to do at work. |
| 9. | My job roles are conflicting and difficult to reconcile. |
| 10. | I have too many roles to juggle. |
| 11. | There is a lack of democracy in my organization. |
| 12. | There is a lack of cooperation and communication between the various departments within my work organization. |
| 13. | It is difficult to work creatively and autonomously in my unit. |
| 14. | There are often various rights disputes in my unit. |
| 15. | My work is simple, low-level repetitive work, meaningless. |
| 16. | I cannot grow from the completion of the tasks of the post. |
| 17. | There are very few opportunities for advancement in my job position. |
| 18. | It's hard to make grades at my job. |
| 19. | There is no sense of achievement in completing tasks. |
| 20. | There is no future in my work. |
| 21. | The difference between giving and receiving is too great, and I feel that the meaning and value of work is low. |
| 22. | My job responsibilities are heavy. |
| 23. | The job requires me to manage my relationship with the leader. |
| 24. | The cultural value orientation of the unit makes me uncomfortable. |
| 25. | The job requires me to adapt to other people's interpersonal style. |
| 26. | At work, I often feel that my energy is not enough and my physical strength is exhausted. |
|  | **Work Adaptability** |
| 1. | I respond appropriately to threats, dangers or emergencies in my life. |
| 2. | When dealing with emergencies, I can quickly analyze various courses of action and take effective action. |
| 3. | In conflict situations, I can be calm and objective in dealing with the problem. |
| 4. | In my role, I can handle high workloads or intense work rhythms. |
| 5. | I can calmly analyze and deal with emergencies at work. |
| 6. | I like to have work pressure, I feel that pressure is what drives my work to be effective. |
| 7. | When I get frustrated at work, I always find a way to fix it instead of putting the blame or blaming others. |
| 8. | I analyze problems thoroughly and find novel, creative solutions from seemingly irrelevant information. |
| 9. | I can generate creative new ideas with unique analysis when the problem situation is complex. |
| 10. | When the resources needed to complete the task are not enough, I will find a way to get new resources. |
| 11. | I can often think of solutions to problems that no one else has found. |
| 12. | I can apply the new technologies and new knowledge I have learned to my work. |
| 13. | Changes in the current and future economic and competitive environment require me to keep learning more and more new knowledge. |
| 14. | I am keen to learn new knowledge, new working methods, new technologies. |
| 15. | I think I can quickly adapt to work activities I haven't been exposed to before. |
| 16. | I can improve the flaws in my work and constantly explore the most effective way to work. |
| 17. | I can get along with others flexibly at work and have a good relationship with other people. |
| 18. | At work, I can listen to and think about the opinions of others, and change my own inappropriate thoughts. |
| 19. | I can deal effectively with people of different personalities at work and build good working relationships with them. |
| 20. | I can see into the behavior of others and adjust my behavior at any time to work more effectively with others. |
| 21. | I take the initiative to understand other departments, the working atmosphere and value orientation of the organization. |
| 22. | I enable myself to act in a way that demonstrates identification with the values, habits and cultures of other groups and organizations. |
| 23. | I can integrate into the values, customs and cultures of different styles. |
| 24. | I can understand the activities of people with different cultural values and maintain positive relationships with them. |
| 25. | I can act effectively even when the information needed to solve a problem is incomplete and the big picture is unclear. |
| 26. | I am open to changing my behavior to accommodate unpredictable work events or situations. |
| 27. | I can effectively adjust work plans and goals at any time according to changing circumstances. |
| 28. | I can focus on improving work performance when the work situation is not very clear. |
| 29. | I am physically able to take on a tough work environment. |
| 30. | I am physically fit enough for my current job role and situation. |
| 31. | I have plenty of energy to adapt to high-paced work tasks. |
|  | **Occupational Health** |
| 1. | I often get restless due to certain problems at work. |
| 2. | I often feel physically exhausted after get off work. |
| 3. | Feeling depressed due to troubles at work. |
| 4. | Annoyed by things around you. |
| 5. | I feel like I'm irritable sometimes. |
| 6. | Feeling nervous about things at work. |
| 7. | I get angry easily with work problems. |
| 8. | Worrying about how I get the job done. |
| 9. | Work is too stressful and I don't get enough rest. |
| 10. | At work, I often have a sense of urgency. |
| 11. | Work takes a heavy toll on my spirit. |
